# The Carpentier-Edwards Perimount Magna mitral valve bioprosthesis: intermediate-term efficacy and durability

**DOI:** 10.1186/s13019-016-0412-4

**Published:** 2016-01-27

**Authors:** Gabriel Loor, Andres Schuster, Vincent Cruz, Aldo Rafael, William J. Stewart, James Diaz, Kenneth McCurry

**Affiliations:** Department of Cardiothoracic Surgery, University of Minnesota, 420 Delaware Street SE, MMC 207, Minneapolis, MN 55455 USA; Department of Cardiology, Cleveland Clinic, Cleveland, USA; Lerner College of Medicine, Cleveland Clinic, Cleveland, USA; Department of Cardiac Surgery, Baylor University Medical Center, Dallas, USA; Department of Thoracic and Cardiovascular Surgery, Cleveland Clinic, Cleveland, USA

## Abstract

**Background:**

The Carpentier-Edwards Perimount Magna mitral valve bioprosthesis (Edwards Lifesciences, Irvine, CA) is a low-profile version of the earlier Perimount valve that uses the ThermaFix process for enhanced calcium removal. The Magna valve has been in use since 2008, yet no publication, until now, has verified its intermediate-term safety and efficacy.

**Methods:**

From 2008 through 2011 (our 4-year study period), 70 Magna valves were implanted in the mitral position at a single institution (the Cleveland Clinic). Echocardiograms were prospectively interpreted. For this study, we reviewed patients’ charts; endpoints included hemodynamic measurements, in-hospital morbidity and mortality, valve-related events, resource utilization, and 5-year survival rates.

**Results:**

The mean patient age was 68 years; 43 % of the patients had New York Heart Association (NYHA) class III or IV disease, and 51.4 % had moderately severe, or worse, mitral regurgitation (MR). For 43 % of the patients, the Magna valve implantation was a reoperation. For 83 %, the Magna valve implantation also included a concomitant cardiac procedure. The median survival rate was 4.7 years and 90 % of patients were free from significant structural valve degeneration at 5 years. Preoperative atrial fibrillation, ischemic MR, intraaortic balloon pump placement, cardiogenic shock, cardiac arrest, and renal failure were associated with increased mortality. Right ventricular systolic pressure decreased from 50 mmHg preoperatively to 40 mmHg postoperatively, according to our matched-pair analysis (*P* = 0.003*)*. Per their final echocardiogram during our study period, 98 % of surviving patients had trivial or no MR, one patient had mild MR, and one patient had severe MR.

**Conclusions:**

Our 5-year experience indicates that the Magna valve offers excellent intermediate-term durability and substantial echocardiographic improvement; its low-profile design make it ideal for reoperations and for concomitant cardiac procedures, including valve replacement.

## Background

Each year, more than 20,000 mitral valve operations are reported to the Society of Thoracic Surgeons database [1]. Despite the increased emphasis on valve repair, at least 30 % of patients with mitral valve disease still undergo valve replacement [2–4]. Reasons include extensive comorbidities, previous valve operations, complex jets, and mitral stenosis [3]. The ideal replacement valve would be durable, would not require anticoagulation, and would be small enough to avoid distortion of the mitral annulus while preserving left ventricular geometry.

A mitral valve bioprosthesis avoids anticoagulation, but is associated with a higher reoperation rate than a mechanical valve. For patients older than 70 years and for patients with a contraindication to anticoagulation, a mitral valve bioprosthesis is the preferred replacement option [1, 5–8]. Several are commercially available and approved by the U.S. Food and Drug Administration (FDA). Among the most popular are the St. Jude Medical Biocor (St. Jude Medical, St. Paul, MN) and the Carpentier-Edwards Perimount mitral valve bioprosthesis (Edwards Lifesciences, Irvine, CA). With both of those valves, the published short- and long-term hemodynamic and clinical data have suggested good outcomes [7, 9–12].

In 2008, Edwards Lifesciences modified that initial Perimount model to provide a lower profile and to enhance removal of calcium-binding sites through its ThermaFix process. This modified model is referred to as the Carpentier-Edwards Perimount Magna mitral valve bioprosthesis, or the Magna valve for short. It protrudes less into the left ventricular outflow tract (LVOT) than its predecessor, so it is appealing for patients with small ventricles undergoing multiple valve procedures or reoperations. It is also predicted to have less structural valve deterioration, thanks to the ThermaFix process.

The Magna valve, building on the proven durability of the initial Perimount model, has now been implanted in centers around the United States. Yet no publication, until now, has verified its short- and intermediate-term safety and efficacy. Herein, we report our 5-year outcomes with the use of the Magna valve in 70 patients implanted between 2008 through 2011 at a single center (the Cleveland Clinic)—including short-term in vivo echocardiographic data, which can be used as a reference for outcomes analyses and for future valve modifications.

## Methods

### Study population

**Fig. 1:**
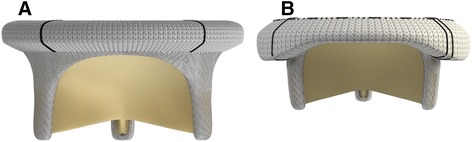
**a**. Original Carpentier-Edwards Perimount design. **b**. Magna valve based on the original design but now with a lower profile and Therma fix processing

For our study, we queried the Cardiovascular Information Registry (Cleveland Clinic), a prospective database approved for use in research by the Institutional Review Board. Included in our study population were patients who underwent mitral valve replacement with the Magna valve from 2008 through 2011 (our 4-year study period) (Fig. 1). Excluded were patients who underwent mitral valve repairs or replacements with other types of valves.

In general the Cleveland Clinic prefers mitral valve repair whenever possible. When replacement is needed, we have primarily used either the St. Jude Medical Biocor or the Carpentier-Edwards Perimount mitral valve bioprosthesis; however, since 2008, our experience with the Magna valve has been gradually accumulating.

After querying the Cardiovascular Information Registry, we reviewed patients’ electronic medical records and entered their clinical data into our own working database for analysis. This database was approved for use by the Cleveland Clinic’s Institutional Review Board, which waived the need for consent from individual patients. Clinical data included demographic variables, comorbidities, operative details, postoperative outcomes, and intermediate-term follow-up results. To confirm preoperative atrial fibrillation, we reviewed the preoperative electrocardiogram. Coronary artery disease was defined as ≥50 % obstruction of a coronary vessel that may or may not have been amenable to bypass. Emergency operations were performed within 24 h after the clinical diagnosis. Carotid artery disease was defined as ≥50 % obstruction, per carotid artery duplex scan findings. Pulmonary hypertension was defined as right ventricular systolic pressure (RVSP) ≥40 mmHg, per echocardiography findings.

### Endpoints

Endpoints were postoperative hemodynamic measurements, in-hospital morbidity and mortality, valve-related events, resource utilization, clinically significant structural valve degeneration and intermediate-term survival rates. To define in-hospital morbidity (perioperative myocardial infarction, respiratory failure, sepsis, renal failure, and neurologic complications), we used the Society of Thoracic Surgeons national Adult Cardiac Surgery database. For surviving patients, we used hospital length of stay as a surrogate marker for resource utilization. Clinically relevant structural valve degeneration was defined as the need for reoperation or severe regurgitation or stenosis preceding death.

### Hemodynamic measurements

For the echocardiography findings, we retrieved the last available transthoracic study from our server for each patient; for all quantifications, we used Syngo software (Siemens AG, Munich, Germany), with interpretations made by two experienced echocardiologists (A.S and W.S.). For the 2D echocardiography analysis of LVOT diameter, left ventricular ejection fraction (EF), and stroke volume (SV), we used the biplane method of disks (modified Simpson’s rule). We also recorded RVSP, peak and mean transmitral gradients (mmHg), left atrial (LA) size, left ventricular internal diameter (LVID), and effective orifice area (EOA).

To calculate the LVOT systolic velocity time integral (VTI), we used the mean of three waveforms acquired by LVOT pulse-wave Doppler; then, to calculate SV, we multiplied LVOT diameter by LVOT VTI. If systolic LVOT VTI was not available, we obtained SV by using the biplane method of disks (modified Simpson’s rule).

To calculate the peak transmitral gradient, we obtained measurements from mitral diastolic continuous-wave Doppler assessment and then used the simplified Bernoulli equation. To calculate the mean transmitral gradients, we obtained measurements from the diastolic waveform VTI. For both the peak and mean measurements, we used the mean of three waveforms.

To calculate the mitral valve area (MVA), we used the pressure half-time (t_1/2_) method (MVA = 220/t_1/2_). To calculate the mitral EOA, we divided SV by the mitral diastolic VTI. To calculate the EOA index, we divided the EOA by body surface area.

To assess the presence and degree of mitral regurgitation, we used the four-chamber view per color flow Doppler imaging, according to the semiquantitative approach recommended by the American Society of Echocardiography. In six of the 70 patients, postoperative transthoracic 2D echocardiograms were not available for analysis.

### Data analysis

Continuous demographic variables are expressed as the mean ± the standard error of the mean (SE); categorical values are expressed as the n (percentage). To compare pre- and postoperative echocardiography findings, we used the paired Student *t* test. To assess intermediate-term survival (in months) after Magna valve implantation, we used the Kaplan-Meier method, with censoring (*N* = 70, with *n* = 16 deaths, 54 censored). To assess freedom from structural valve degeneration we used the Kaplan-Meier method (*N* = 70, with *n* = 2 events, 68 censored). To analyze the relationship between dichotomous variables and survival, we used the 2-tailed Fisher exact test.

## Results

Preoperative demographic and clinical characteristics are reported in Table 1. The mean age was 68 years (range, 29 to 88). Of the 70 patients, 30 (43 %) had New York Heart Association (NYHA) class III or IV disease. The Magna valve implantation was considered an emergency operation in 3 (4.3 %) of the patients; 9 (12.8 %) were in cardiogenic shock preoperatively. Additionally, 36 (51.4 %) of the patients had moderately severe, or worse, mitral regurgitation; 21 (30 %), severe mitral stenosis. In terms of previous surgery, 30 (42.8 %) of the patients had undergone at least 1 previous cardiac operation; 19 (27.1 %), a previous mitral valve repair or replacement.

**Table 1:** Patient characteristics (*N* = 70)^a^

*Demographics*	
Age, years	68 ± 1.6
Gender	
Men	36 (51 %)
Women	34 (49 %)
*Preoperative clinical and laboratory values*	
BMI,^b^ kg/m^2^	26.5 ± 0.7
Hematocrit, %	35.8 ± 0.7
Creatinine (mg/dl)	1.6 ± 0.2
*Disease acuteness*	
NYHA functional class	
I or II	40 (57 %)
III or IV	30 (43 %)
Emergency operation	
Yes	3 (4.3 %)
No	67 (96 %)
IABP use	
Yes	3 (4.3 %)
No	67 (96 %)
*Cardiac comorbidities*	
Coronary artery disease	21 (30 %)
EF, %	55 ± 1.2
Moderately severe, or worse, MR	36 (51 %)
Severe MS	21 (30 %)
History of heart failure	
Yes	22 (31 %)
No	48 (69 %)
Previous MI	
Yes	10 (14 %)
No	60 (86 %)
Preoperative cardiogenic shock	9 (13 %)
Yes	
No	61 (87 %)
History of peripheral artery disease	
Yes	4 (5.7 %)
No	66 (94 %)
Previous cardiac operation	
Yes	30 (43 %)
No	40 (57 %)
Previous mitral valve repair or replacement	
Yes	19 (27 %)
No	51 (73 %)
Preoperative AF	
Yes	19 (27 %)
No	51 (73 %)
History of hypertension	
Yes	53 (76 %)
No	17 (24 %)
*Noncardiac comorbidities*	
Previous stroke	
Yes	8 (11 %)
No	62 (89 %)
History of carotid artery disease^b^	
Yes	26 (37 %)
No	43 (61 %)
History of smoking	
Yes	29 (41 %)
No	41 (59 %)
History of COPD	
Yes	11 (16 %)
No	59 (84 %)
History of DM	
Yes	17 (24 %)
No	53 (76 %)
History of renal disease	
Yes	20 (29 %)
No	50 (71 %)

*BMI* body mass index, *NYHA* New York Heart Association, *IABP* intraaortic balloon pump, *EF* ejection fraction, *AF* atrial fibrillation, *MI* myocardial infarction, *COPD* chronic obstructive pulmonary disease, *DM* diabetes mellitus, *MR* mitral regurgitation, *MS* mitral stenosis

^a^Continuous variables expressed as mean ± standard error of the mean (SE); categorical variables, as n (percentage)

^b^Data available for only 69 patients

Operative factors and findings are summarized in Table 2. Of the 70 patients, 69 (98.6 %) underwent a full sternotomy. The most common approach to Magna valve implantation was transeptal with subvalvular leaflet preservation. The most common Magna valve size was 29 mm, followed by 25 mm. Mitral annular calcification was the most common operative finding, followed by degenerative disease. Most patients had at least 1 additional procedure performed at the time of the Magna valve implantation.

**Table 2:** Operative factors and findings (*N* = 70)^a^

*Operative factors*	
Indication	
MR	49 (70 %)
MS	19 (27 %)
Endocarditis	10 (14.2 %)
Attempted repair	
Yes	7 (10 %)
No	63 (90 %)
Minimally invasive Magna valve implantation	
Yes	1 (1.4 %)
No (i.e., full sternotomy)	69 (99 %)
Technique for Magna valve implantation	
Transseptal approach	49 (70 %)
Left atriotomy	21 (30 %)
Subvalvular leaflet preservation	56 (80 %)
Magna valve size^b^ (mm)	28 ± 0.3
25	18 (26 %)
27	16 (23 %)
29	19 (27 %)
31	16 (23 %)
33	1 (1.4 %)
*Findings*	
Rheumatic disease	12 (17 %)
Myxomatous disease	4 (5.7 %)
Ruptured chordae	5 (7.1 %)
Degenerative disease	28 (40 %)
Vegetations	10 (14 %)
MAC	38 (54 %)
Ischemic MR (posterior restriction)	3 (4.3 %)
Concomitant procedures	
Any	58 (83 %)
CABG	6 (8.6 %)
AVR, TVR, ASD repair, and/or myectomy	33 (47 %)
CABG + AVR, TVR, ASD repair, and/or myectomy	11 (16 %)
AVR	24 (34 %)
Triple valve procedures	6 (8.6 %)
Antiarrhythmic procedure (Cox maze procedure, PVI, and/or LAAL)	7 (10 %)

*mm* millimeters, *MR* mitral regurgitation, *MS* mitral stenosis, *MAC* mitral annular calcification, *CABG* coronary artery bypass grafting, *AVR* aortic valve replacement, *TVR* tricuspid valve repair, *ASD* atrial septal defect, *PVI* pulmonary vein isolation, *LAAL* left atrial appendage ligation

^a^Continuous variables expressed as mean ± standard error of the mean (SE); categorical variables, as n (percentage)

^b^Data available for only 68 patients

Thirty eight patients had at least some MAC documented in the operative note. Moderate to severe MAC was noted in 29 of these cases (57 %). MAC involved the anterior annulus in 22 cases (57 %) and the posterior annulus in 36 cases (94 %). In 17 cases (44 %) the MAC was left undisturbed.

Twelve cases (31 %) required at least some debridement and nine cases (23 %) required extensive debridement. Annular reconstruction with a patch was used to avoid AV rupture if adipose tissue was visible on the ventricular side of the annulus after debridement. Three cases (8 %) used a bovine pericardial patch and 1 case (3 %) used a felt patch. Only one case required an additional bypass run for mild-moderate perivalvular regurgitation to reinforce a separation along the anterior rim. The rest had either no or trivial MR at the conclusion of the replacement.

Postoperative complications are detailed in Table 3. We noted five in-hospital deaths, for a mortality rate of 7.4 % in the immediate postoperative period. For the remaining 65 survivors, the mean hospital length of stay was 12 days (range, 4 to 42). Two patients (2.9 %) suffered a stroke within 30 days. The most common rhythm disturbance was atrial fibrillation (71 %). In all, 6 (8.5 %) of the patients developed renal failure requiring dialysis; 3 (4.2 %) suffered an in-hospital cardiac arrest.

**Table 3:** Postoperative course and complications (*N* = 70)^a^

Hospital LOS,^b^ days	11.6 ± 0.8
Cardiogenic shock^c^	6 (8.6 %)
IABP use	2 (2.9 %)
Tracheostomy	4 (5.7 %)
Any renal failure	17 (24 %)
Renal failure requiring dialysis	6 (8.6 %)
Reoperation for bleed	4 (5.7 %)
AF	50 (71 %)
Heart block	19 (27 %)
Transient	16 (23 %)
Permanent pacer	3 (4.2 %)
Wound infection	1 (1.4 %)
Cardiac arrest	3 (4.3 %)
In-hospital death	5 (7.1 %)
Stroke	2 (2.9 %)
Valve-related complications	
Thrombosis	0 (0 %)
Dehiscence	0 (0 %)
LVOTO^d^	1 (1.4 %)
Vegetations	0 (0 %)
AV groove disruption	1 (1.4 %)

*AF* atrial fibrillation, *AV* atrioventricular, *IABP* intraaortic balloon pump, *LOS* length of stay, *LVOTO* left ventricular outflow tract obstruction

^a^Continuous variables expressed as mean ± standard error of the mean (SE); categorical variables, as n (percentage)1

^b^Data available for only 65 patients

^c^Data available for only 69 patients

^d^Required reoperation at 2 years

**Fig. 2:**
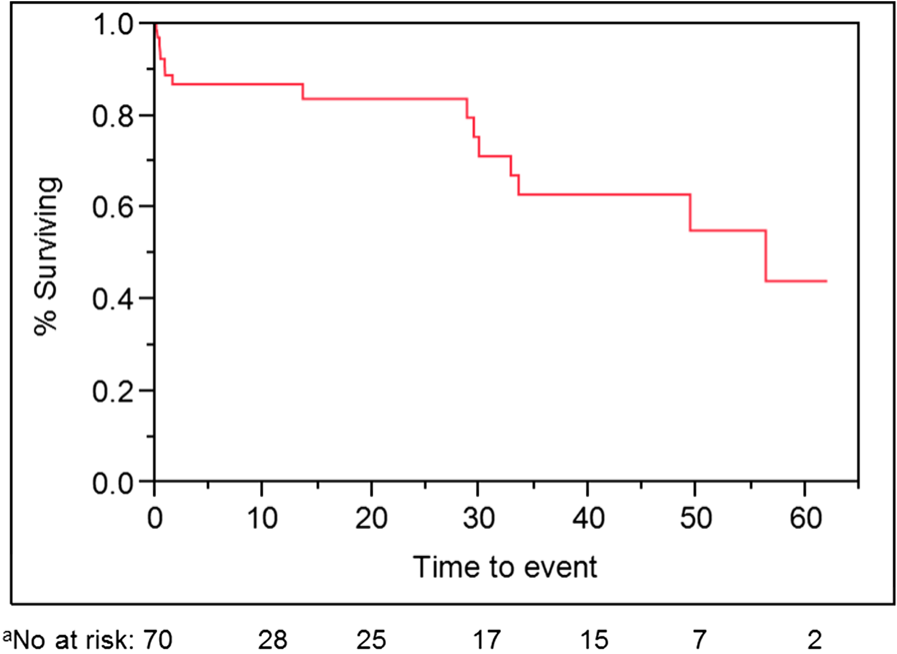
Kaplan-Meier non-parametric estimate of all-cause mortality based on the observed survival in patients with the Carpentier-Edwards Magna valve in the mitral position with censoring (*N* = 70, with *n* = 16 deaths, 54 censored). X-axis represents time after valve implant, in months. ^a^Number at risk for each 10 month interval beginning with n of 70

The average clinical follow-up was 16 months (range, 0–62 months). The 2- and 5-year survival rates were 84 and 40 % respectively. The median survival was 4.7 years (Fig. 2). Several dichotomous perioperative factors were associated with nonsurvival, including preoperative atrial fibrillation, ischemic mitral regurgitation, use of an intraaortic balloon pump (IABP), cardiogenic shock, cardiac arrest, and renal failure (Table 4). Interestingly, RVSP ≥40 mmHg was associated with a higher survival rate on univariate analysis (*P* = 0.02) suggesting, at minimum, that no penalty was incurred for Magna valve implantation in patients with moderate pulmonary hypertension.

**Table 4:** Factors associated with increased mortality (*N* = 70)^a^

	Survivors (*n* = 62)	Nonsurvivors (*n* = 8)	*P* ^b^
AF	14 (23 %)	5 (63 %)	0.02
Ischemic MR	1 (1.6 %)	3 (38 %)	0.03
IABP use	0 (0 %)	2 (25 %)	0.01
Cardiogenic shock	2 (3.2 %)	4 (50 %)	0.001
Cardiac arrest	1 (1.6 %)	2 (25 %)	0.03
Any renal failure	15 (24 %)	6 (75 %)	0.007

*AF* atrial fibrillation *IABP* intraaortic balloon pump, *MR* mitral regurgitation

^a^ Continuous variables expressed as mean ± standard error of the mean (SE); categorical variables, as n (percentage)

^b^ Relationship between dichotomous variables and survival analyzed with 2-tailed Fisher exact tests

Pre- and postoperative echocardiography findings are compared in Table 5. The mean follow-up time for postoperative echocardiography was 5 months (range, 0.3 to 50 months). Per their final echocardiogram during our study period, 98 % of surviving patients had trivial or no mitral regurgitation, one patient had mild mitral regurgitation and one patient had severe mitral regurgitation. No short term valve related events such as vegetations, dehiscence, degeneration, stenosis, or thrombosis were documented on any of the postoperative echocardiograms. However, one patient had obstruction of the LVOT by a strut requiring a reoperation at 2 years.

**Table 5:** Pre- and postoperative echocardiography findings (*N* = 70)^a^

Left ventricular EF^b^	
Preoperative, %	54.8 % ± 1.25 %
Postoperative, %	52.3 % ± 1.67 %
Difference, %	−2.50 % ± 1.57 %
*P*	0.1160
RVSP^c^	
Preoperative, mmHg	50.1 ± 3.23
Postoperative, mmHg	39.8 ± 2.18
Matched-pair difference, mmHg	−10.3 ± 3.23
*P*	0.0025
LA size^d^	
Preoperative, cm^2^	5.557 ± 0.560
Postoperative, cm^2^	4.641 ± 0.113
Matched-pair difference, cm^2^	−0.915 ± 0.558
*P*	0.1158
LVID^e^	
Preoperative, cm	4.705 ± 0.883
Postoperative, cm	4.598 ± 0.863
Matched-pair difference, cm	−0.107 ± 0.098
*P*	0.116

*cm* centimeters, *EF* ejection fraction, *LA* left atrial, *LVID* left ventricular internal diameter, *mmHg* millimeters of mercury, *RVSP* right ventricular systolic pressure

^a^Continuous variables expressed as mean ± standard error of the mean (SE)

^b^Both pre- and postoperative data available for only 64 patients

^c^Both pre- and postoperative data available for only 49 patients

^d^Both pre- and postoperative data available for only 46 patients

^e^Both pre- and postoperative data available for only 58 patients

**Fig. 3:**
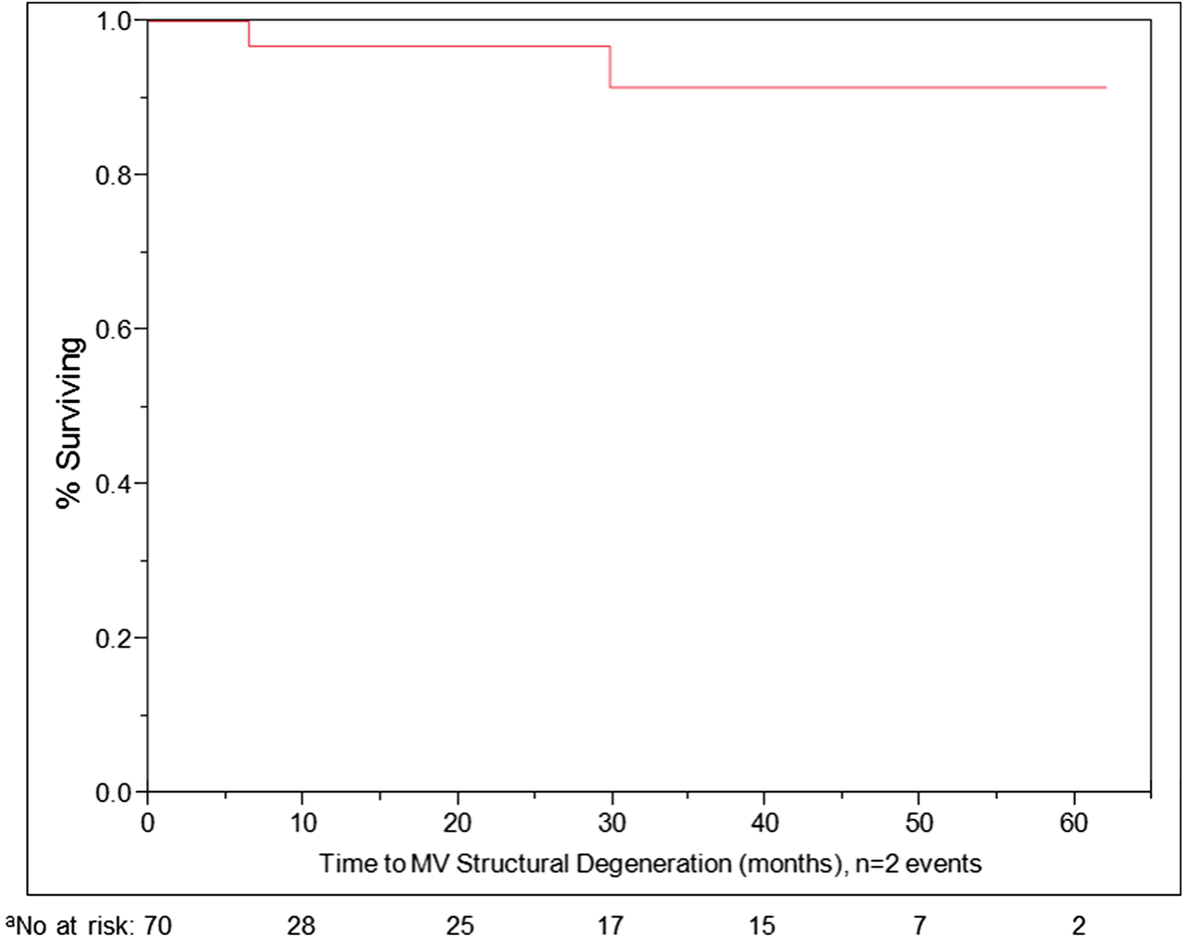
Kaplan-Meier non-parametric estimate of freedom from structural valve degeneration, as defined by a need for reoperation due to valve dysfunction preceding death, in patients with the Carpentier-Edwards Magna valve in the mitral position (*N* = 70, with *n* = 2 events, 68 censored). X-axis represents time after valve implant, in months. ^a^Number at risk for each 10 month interval beginning with n of 70

Two patients had evidence of severe structural valve degeneration on follow-up (Fig. 3). In one, severe prosthetic MR was present at 2 years and the patient died before a reoperation. In the other patient, severe mitral calcification was noted during a redo AVR 6 months after the initial Magna valve implantation. The postoperative EF decreased by a mean of 2.5 % across matched pairs, although the difference was not statistically significant (Table 5). We observed a trend toward a reduction in LA size and in LVID.

**Fig. 4:**
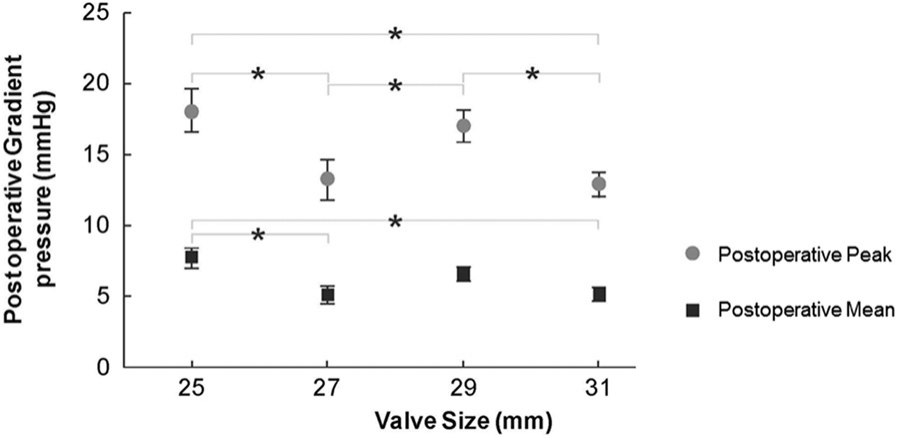
Postoperative peak (*gray circles*) and mean (*dark boxes*) transmitral gradients (in millimeters of mercury) per echocardiography by Magna valve size (in millimeters). Asterisks = means that are significantly different (*P* < 0.05)

**Fig. 5:**
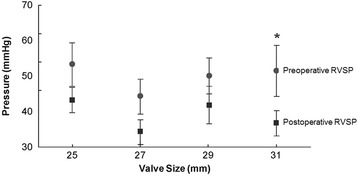
Preoperative (*gray circles*) and postoperative (*dark boxes*) right ventricular systolic pressure (RVSP) (in millimeters of mercury) per echocardiography by Magna valve size (in millimeters). Asterisks = pre- and postoperative matched pairs that are significantly different (*P* < 0.05)

**Fig. 6:**
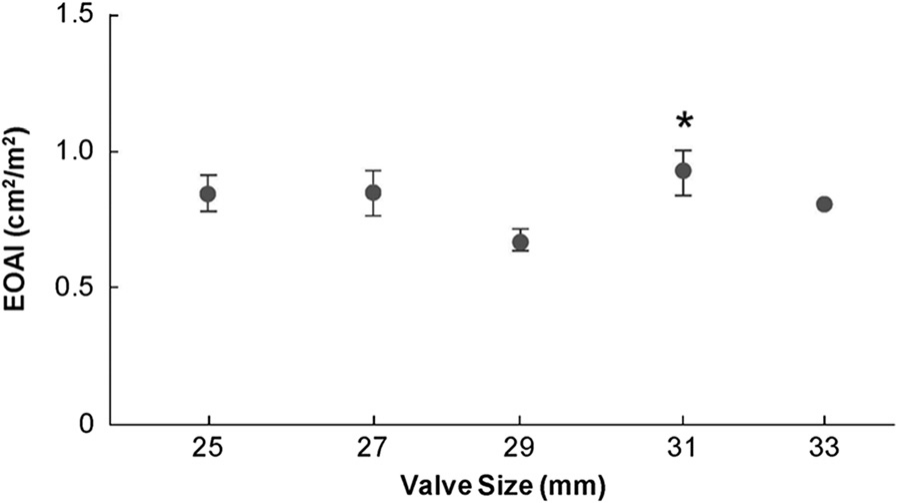
Effective Orifice Area Index (EOAI) across various valve sizes. The *asterisk* denotes a significant difference between the 31 and 25 mm EOAI

The peak transmitral gradient decreased as the Magna valve size increased (Fig. 4). The same was true for the mean transmitral gradient. However, we noted no statistically significant differences in the mean gradient between the two most commonly employed valve sizes (29 and 25 mm), suggesting that even a small Magna valve was capable of yielding a low mean gradient. Although we noted a trend toward lower RVSP values for each Magna valve size, the only statistically significant decrease was with 31 mm (Fig. 5). The Effective Orifice Area Index (EOAI) did not vary significantly between valve sizes except for the 31 mm valve which had a statistically higher value than the 25 mm valve (Fig. 6).

## Discussion

Our 5-year study is the first to objectively evaluate the hemodynamic and clinical outcomes with the Magna valve, a modification of the earlier Carpentier-Edwards Perimount mitral valve bioprosthesis. We found excellent intermediate-term durability with this versatile design, which is well suited for complex situations; even at the lowest valve sizes, this new model provided patients with excellent hemodynamics.

Most of the patients in our series had extensive comorbidities, a high NYHA class, severe valve disease, and previous cardiac operations, many of which involved the mitral valve. Thus, our patients were typical of those undergoing mitral valve replacement in the current era [1, 4, 13]. While the survival rate at 2 years of 84 % was excellent, the survival rate of 40 % at 5 years was commensurate with this cohorts’ extent of illness. For comparison, the St. Jude Medical Biocor valve series from Rizzoli et al. noted a 5-year survival rate of 54 % [12]. The 15 year experience with the original Perimount bioprosthesis showed a 5 year survival of 76 %, although their cohort had fewer comorbidities and less reoperations than observed in the current study [7]. We also observed significant postoperative morbidity, including renal failure, cardiogenic shock, and respiratory failure. One patient suffered a death due to AV groove disruption which was related to severe MAC rather than the Magna valve itself. One reoperation was required at 2 years due to LVOT obstruction from a strut. These cases highlight the need for caution even with this low-profile design. The freedom from structural valve degeneration of 90 % was excellent and compares favorably with that observed in other series [7, 12]. One reoperation was due to structural valve degeneration at 6 months and one patient died due to severe MR at 2 years without a reoperation.

We identified several factors associated with increased mortality in our patients, including atrial fibrillation, cardiogenic shock, IABP use, cardiac arrest, and ischemic mitral regurgitation. Wang et al. also found significantly increased mortality in patients with preoperative atrial fibrillation, but they found improved survival with a concomitant Cox maze and LA ligation [14]. In our series, despite the 27 % incidence of preoperative atrial fibrillation, only 10 % of our patients underwent an antiarrhythmic procedure—perhaps a reflection of the length and complexity of the procedures, along with the frailty of the tissues, which discouraged ancillary procedures that were not absolutely necessary in these older patients.

De Bonis et al. recently showed that ischemic mitral regurgitation in sicker patients was associated with lower survival when the mitral valve was replaced rather than repaired [15]. Conversely, Gillinov et al. found no difference in survival between repair and replacement in patients with a higher NYHA class and complex regurgitant jets [4]. This is consistent with the randomized controlled prospective trial of Acker and colleagues that showed no survival benefit for repair over replacement and greater durability with valve replacement [16]. We believe it is reasonable to use the Magna valve for ischemic mitral regurgitation, although repair should be considered in appropriate candidates.

What are the advantages of a lower-profile bioprosthesis? Patients receiving a mitral prosthesis have a natural reduction in the LVOT [17]. In addition, the struts of a bulky bioprosthetic can protrude into the LVOT, causing a clinically significant increase in gradients [18]. A smaller, lower-profile valve reduces such concerns. Reoperative or multiple-valve procedures in patients with mitral annular calcification or degenerative disease pose additional restrictions on the prosthesis size. The exposure in such procedures is challenging; the mitral annulus and left ventricular cavities are often restricted. Most patients in our series underwent a full sternotomy, with transseptal mitral exposure and with implantation of a small Magna valve (25 to 29 mm). In addition, in patients with ischemic mitral regurgitation, the Magna valve’s low-profile design is more likely to allow preservation of the subvalvular apparatus which may be associated with improved survival [19, 20]. In our series, 80 % of patients had preservation of some portion of the subvalvular apparatus, with no reported outflow obstruction.

The mean gradients achieved with the Magna valve in our series compared favorably with those reported in previous publications on other mitral bioprostheses [10–12]. Our lowest gradients were achieved with the 27 and 31-mm Magna valves, although the gradients achieved with the 25-mm Magna valve were only slightly higher. Thus, the 25-mm valve size is a reasonable option for patients with a constrained annulus or small ventricle, especially when left ventricular outflow tract obstruction (LVOTO) is a concern. While the low profile design may reduce the incidence of LVOTO, care must still be taken to keep the struts out of the way of the LVOT as one case in this series did demonstrate LVOTO resulting in redo-replacement.

The valve size of 25 was the second most common size used in this series which suggests small working annuluses. This could be explained by variations in patient anatomy or surgical technique, high rate of MAC with limited debridement, and/or the presence of reoperations. We tend to use either inverting (ventricular to atrial) or everting (atrial to ventricular) suture techniques depending on anatomy and ease of placement. We favor inverting sutures in the setting of MAC which places the sewing ring on the atrial side of the mitral annulus. If everting sutures are used then the valve may sit slightly intra-annular. We will infrequently construct a neo-inner annulus as described by Di Stefano and colleagues to avoid debridement in the setting of severe MAC [21]. The presence of favorable mean gradients suggests that the range of Magna sizes can accommodate the operator’s preference and/or patient’s anatomy.

The favorable hemodynamic profile of the Magna valve is further supported by the significant reduction we observed in RVSP. It is anticipated that a persistent reduction in RVSP could correlate with improved long-term survival, but we did not have a large enough sample size or long enough follow-up to definitively show any correlation [22]. The suggestion that even smaller Magna valve sizes produce favorable hemodynamics is supported by the fact that we saw no appreciable difference in EOA index across valve sizes, except for the 31-mm valve.

Our study supports the use of the Magna valve as a replacement option, but we acknowledge several important limitations. First, echocardiographic follow-up was not as standardized as it is for repairs and not all patients had echos at their longest follow-up interval. Echocardiography was generally obtained if a clinical indication was met. Thus, our review of structural valve degeneration was limited to the causes of death, reoperation and the last available echo prior to these events. The Magna valve design is based on the previous Perimount model, which is associated with 14 years’ worth of data showing freedom from explantation and from structural valve deterioration, so we are confident that the newer model will do at least as well. Additionally, the prospective evaluation of the echocardiograms by two experienced cardiologists was crucial as it standardized the valve’s performance measures. But it was limited to a mean follow-up of 5 months with a broad range of intervals. We elected to evaluate the last echos to avoid an unfair advantage from early postoperative performance. We noted a high interobserver variability for the calculation of the EOA index in our series, making it difficult to reach conclusions about the actual value for any given Magna size. However, we were able to make statistical comparisons for changes in the EOA index across valve sizes. Our study was underpowered for a multivariate analysis, although trends were established by univariate measures.

The peak gradient for the 25, 29 and 31 mm valves gradually decreased as expected. However, the peak was higher for the 29 mm valve than the 27 mm valve. Similarly, the 25, 27 and 31 mm mean gradients gradually decreased as expected. But the mean for the 29 and 25 mm valves were not statistically different. Peak and mean gradients can be affected by various transient factors such as cardiac output, heart rate, and volume status. In addition, the patient population was heterogeneous and we did not have an equal distribution of patients throughout the various valve sizes. Thus, we can conclude that the peaks and means decreased in our series with larger valve sizes as expected but we cannot give definitive estimates of these relative gradients that would translate to a broader population.

## Conclusions

In conclusion, this is the first published report on outcomes after implantation of the Magna valve. Modifications are regularly made to valves; it is important that at least short-term results are made available. In our 5-year study, we found excellent freedom from structural valve degeneration, excellent short term survival, good hemodynamics at even the lowest valve sizes, substantial echocardiographic improvement, and minimal valve-related events. The Magna valve’s low-profile design and ThermaFix process make it ideal for patients who have small ventricles, extensive comorbidities, or complex valve disease, as well as for those who need reoperations and concomitant cardiac procedures, including valve replacement.
